# The Effects of Decreasing Maternal Anxiety on Fetal Oxygenation and Nucleated Red Blood Cells Count in the Cord Blood

**Published:** 2014-06

**Authors:** Zahra Masoudi, Marziyeh Akbarzadeh, Farideh Vaziri, Najaf Zare, Mani Ramzi

**Affiliations:** 1 Department of Midwifery, School of Nursing and Midwifery; 2 Community Based Psychiatric Care Research Center; 3 Department of Biostatistics, School of Medicine, Infertility Research Center; 4 Division of Hematology, Oncology and Stem Cell Transplantation, Hematology Research Center, Shiraz University of Medical Sciences, Shiraz, Iran

**Keywords:** Acupressure; Anxiety; Delivery Outcome; Doula

## Abstract

***Objective:*** Vasoconstriction during anxiety reduces fetal oxygenation and leads to hypoxia. Hypoxia in turn results in increase of the number of nucleated red blood cells (NRBCs) in the cord blood. The present study aimed to assess the effect of decreasing maternal anxiety on fetal oxygenation and NRBCs count in the cord blood.

***Methods:***. In this study, 150 women were randomly divided into two intervention groups [supportive care and acupressure in BL32 (bladder) acupoint] and a control group (hospital routine care). The infants' cord blood was investigated regarding the number of NRBCs and the intensity of hypoxia after birth. Then, the data were entered into the SPSS statistical software (v. 16) and analyzed using ANOVA, Chi-square test, and logistic regression analysis.

***Findings*** ***:*** The significant difference was found between the two groups regarding the number of NRBCs counted in the peripheral blood smear (*P*<0.001). Besides, a significant relationship was observed between the length of the first and second stages of labor and the number of NRBCs in the cord blood (*P*=0.01). Also, a significant association was observed between the type of delivery and the number of NRBCs in the cord blood in both intervention (*P*<0.001) and control groups (*P*=0.03).

***Conclusion:*** Doula supportive care and acupressure at BL32 point reduced the length of labor stages as well as the anxiety level. Also, nucleated red blood cells were less in the 2 groups of intervention than in control group. Regarding the fact that nucleated red blood cells cannot be the only factor for hypoxia predicting, for affirmation of this theory study with higher sample size and survey of mothers at high risk are needed.

## Introduction

Delivery is an important phenomenon and may be one of the most painful and stressful events that mothers experience during their lifetime^[^^1^^]^. The hormones, such as catecholamines, cortisol, epinephrine, and beta-endorphins, which are secreted in response to tension and anxiety, interfere in the progress of cervical dilatation. On the other hand, they influence the uterine smooth muscles and reduce the contractile power of the uterus as well as its efficiency in the delivery process eventually lengthening the delivery, increasing the pain, and leading to anxiety^[^^2^^]^. During long-term anxiety, the autonomous nervous system is stimulated leading to an increase in the contraction of the smooth muscles of the arterial system. This subsequently reduces the uteroplacental blood flow as well as oxygenation to the uterus, leads to fetal hypoxia, and increases abnormal fetal heart rates^[^^3^^-^^5^^]^. Intrauterine hypoxia is an important risk factor of infant mortality and abnormal outcomes during infancy which causes problems in 5-10% of pregnancies^[^^6^^,^^7^^]^. Recently, counting nucleated red blood cells (NRBCs) in every 100 white blood cells (WBCs) in infants' umbilical venous blood is considered as a sign of prenatal hypoxia. Each hypoxia event leads to a compensatory fetal reaction increasing hematopoiesis and entrance of immature red blood cells into the fetal blood flow level of which is associated with prenatal hypoxia^[^^8^^]^. Thus, the relationship between chronic stress and delivery outcomes, shows the necessity for intervention in order to reduce it^[^^9^^]^. In general, non-pharmacological methods are among the most effective methods of coping with stress and delivery pain. One of these methods involves continuous support of the mother by the doula^[^^10^^]^. Hung conducted a semi-experimental study on the effect of a supportive companion during labor on the delivery outcomes. The study showed that supporting the women during labor was effective in improving the uterine dysfunction and reduced the length of labor as well as the cesarean rate. Besides, the supported women felt less exhausted and were more satisfied after the delivery^[^^11^^]^. Teixeira et al (1991) revealed a relationship between the mother's anxiety score and increase in the vascular resistance of uterine arteries^[^^12^^]^. Labor anxiety and release of cortisol and catecholamines may lengthen the labor, decrease the placental blood flow, and lead to fetal hypoxia^[^^13^^]^.

 Acupressure is another non-pharmacological intervention which is used in order to decrease anxiety. Acupressure is an acupuncture branch in which finger pressure is used instead of needles^[^^14^^]^. Studies have shown that acupuncture is effective in releasing oxytocin from the pituitary gland and stimulates the secretion of oxytocin which directly stimulates the uterine contractions leading to beginning and progress of the delivery and reduction of the labor length^[^^15^^,^^16^^]^. In general, various acupuncture points are used to induce and control the delivery and BL32 acupoint [Shang Liao (urinary bladder or bladder meridian)]^[^^17^^]^ was used in the present investigation. In this study, acupressure which is a simple, non-invasive method and doula supportive care was used in order to reduce the mothers' pain and anxiety during labor and prevent undesirable fetal outcomes, such as hypoxia. Thus, the present study aims to assess the effects of decreasing maternal anxiety on fetal oxygenation and f NRBCs count in the cord blood.

## Subjects and Methods

This randomized clinical trial was conducted in the delivery ward of the selected educational center of Shiraz University of Medical Sciences (Shoushtari Hospital) in 2012. Considering d=5, α=0.05, 1-β=0.90, SD=7, and the following formula, a 150-subject sample size (50 subjects in each group) was determined for the study: 



n=2(Z1-α2+Z1-β)2SD2d2

Then, the subjects were selected through simple random sampling and were divided into supportive care, acupressure, and control groups using stratified block randomization. In doing so, a number was randomly selected from the table of random numbers and the researcher moved toward the right or left column or row and wrote the 5 digit numbers down. Since the participants were divided into 3 groups in this study, 3-therapy method was used and classification was performed as follows: A: supportive care group, B: acupressure group, and C: control group. Accordingly, ABC: 1, ACB: 2, BAC: 3, BCA: 4, CAB: 5, and CBA: 6. It should be noted that numbers 0, 7, and 9 were ignored.

 The inclusion criteria of the study were first or second pregnancy, single and term pregnancy, vertex presentation, spontaneous beginning of the delivery process, 3-4 cm cervical dilatation, being 18-35 years old, and having at least middle school degree. The exclusion criteria of the study were: suffering from any physical or mental problems, preeclampsia, having a history of smoking, maternal diabetes, Rh incompatibility, oligohyd-ramnios, and thick meconium,. 

 The study data were collected using a questionnaire including demographic, clinical, and pregnancy information and an observation form including the information about the labor stages and the laboratory results. Also, Spielberger's questionnaire was employed in order to assess the anxiety level. This questionnaire includes 40 questions in two 20-item scales. The first 20 questions measure the state anxiety which is defined as a feeling of worry and tension resulting from situational stress. Yet, the second 20 questions assess the trait anxiety which refers to individual differences in the tendency toward evaluating the situations as threatening or dangerous^[^^18^^]^. In Iran, Aghamohammadi et al (2007) used this questionnaire on 150 patients undergoing surgery and reported its reliability as 97%^[^^19^^]^ which is the basis for the present study. The supportive care and acupressure groups completed this questionnaire before and after the intervention. In the control group, on the other hand, the questionnaire was completed at the beginning of the active phase of the labor and the end of the first stage.

 In the first group, the researcher as the doula was beside the mother from her entering the department. She reassured the mother, encouraged her, and gave her correct and appropriate information which led to the mother's tranquility, understanding the origin of pain, developing a positive attitude toward the pain experience, and increase in her cooperation in the delivery progress.

**Fig. 1:**
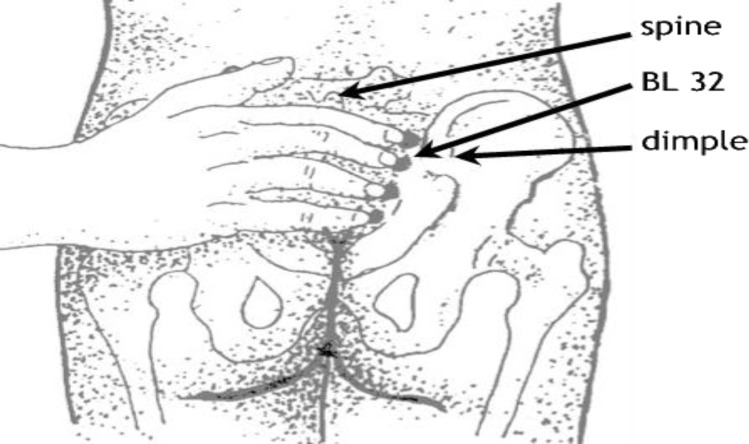
Location of the BL 32 acupoint

 In the second group, in 3-4 and 7-8cm dilatation, the mother was placed in a proper position and BL32 point was pressed. This point lays approximately one index finger length above the top of the buttock crease, approximately one thumb width either side of the spine (Fig. 1)^[^^20^^]^. The pressure was continuously and gently applied by both thumbs for 20 minutes. It was applied by the beginning and stopped at the end of the contractions. On the other hand, the control group only received the department's routine care and underwent no interventions.

 After birth, the infants' cord was double-clamped before or simultaneous with the first breath. In order to facilitate the sampling, the blood was directed toward the cord by the fingers so that the umbilical cord vessels were full of blood. Then, in order to count the total number of the blood cells (CBC diff), 2cc cord blood was poured into a bottle containing Ethylen Diamine Tetracetic Acid (EDTA) and transferred to the hospital laboratory for hematological analysis. After preparing and staining the peripheral blood smear, the number of NRBCs was determined per 100 WBCs and the results were recorded in the related form. Finally, the data were entered into the SPSS statistical software (v. 16) and analyzed using Chi-square, one-way ANOVA, LSD, and logistic regression analysis. *P*<0.05 was considered as statistically significant.

***Findings***

The study results revealed no significant difference between the study groups regarding the demographic variables including mother's age, level of education, occupation, gestational age, and number of pregnancies. In addition, the results of one-way ANOVA showed no significant difference between the three groups regarding the anxiety score before the intervention (*P*=0.4).

**Table 1:** The mothers’ mean score of anxiety (state and trait) before and after the intervention in the three groups

**Anxiety assessment time**	**Supportive care (n=50)** **Mean (SD)**	**Acupressure (n=50)** **Mean (SD)**	**Control (n=50) ** **Mean (SD)**	***P*** **. value**
**Before intervention**	56.8 (2.6)	57.6 (3.6)	57.6 (2.2)	0.4
**After intervention**	35.5 (5.8)	37.5 (9.1)	69.8 (6.6)	<0.001
**Changes in anxiety score** *	-20.5 (8.0)	-20.0 (7.2)	11.1 (9.5)	<0.001

SD: standard deviation

* Anxiety scores after intervention-Anxiety scores before intervention

After the intervention, however, the anxiety mean score was higher in the control group compared to the supportive care and acupressure groups and the difference was statistically significant (*P*<0.001) (Table 1).

 According to the results of Chi-square test regarding the distribution of NRBCs in the peripheral blood smear, the control group showed 68% and 66% difference with the supportive care and acupressure groups, respectively which were statistically significant (*P*<0.001). In all the three study groups, 41.3% (0-10) of NRBCs of the blood cord were observed in the peripheral blood smear. Among the 9 cases with NRBCs in the supportive care group, 14% had 1 and 4% had 2 NRBCs. Also, among the 10 cases with NRBCs in the acupressure group, 10% had 3 and 2% had 1 NRBC. In the control group, on the other hand, among the 43 cases with NRBCs, 18% had 2 and 2% had 10 NRBCs. Overall, a larger number of NRBCs were detected in the peripheral blood smear in the control group compared to the two intervention groups (Table 2).

 Using logistic regression analysis and eliminating the effect of group, a significant relationship was observed between the length of the first and second labor stages and the frequency of NRBCs in the cord blood (*P*=0.01) (OR=1.01). In all the three groups, the mean length of the first and second stages of labor was higher in the cases with NRBCs detected in their peripheral blood. In the control group, the mean length of labor in the cases with NRBCs detected in their peripheral smears was 190.0 and 175.2 minutes higher in comparison to the supportive care and acupressure groups, respectively. Among these cases, the highest and lowest mean length of labor was related to the control and supportive care groups, respectively (Table 3). 

 Based on the results of Chi-square test, a larger number of NRBCs were detected in the peripheral smears in cesarean deliveries. This showed a significant relationship between the type of delivery and the number of NRBCs in the cord blood in both the intervention groups (*P*<0.001) and the control group (*P*=0.03) (Table 4). Moreover, the highest number of NRBCs was observed in cesarean deliveries and this measure in the intervention groups was 14.3% lower in comparison to the control group. In natural vaginal delivery also, the number of cases without NRBCs was 62.7% higher in the intervention groups compared to the control group.

## Discussion

The findings of the present study confirmed the effectiveness of applying pressure at BL32 point and psychologically supporting the mother during labor on reduction of maternal anxiety and improvement of fetal oxygenation.

**Table 2:** Comparison of distribution of cord blood nucleated red blood cells in the intervention and control groups

**NRBC in cord blood count, ** **peripheral smear**	**Supportive care (n=50)** **n (%)**	**Acupressure (n=50)** **n (%)**	**Control (n=50)** **n (%)**	**Total (n=150)** **n (%)**
**Unobserved**	41 (82)	40 (80)	7 (14)	88 (58.7)
**Observed**	9 (18)	10 (20)	43 (86)	62 (41.30
**Rang**	0-2	0-3	0-10	0-10

*P. *value<0.001; NRBC: Nucleated red blood cells

**Table 3:** The relationship between duration of the first and second stages of labor and frequency of cord blood nucleated red blood cells in the intervention and control groups

**NRBC in cord blood count, ** **peripheral smear**	**Supportive care (n=50)** **Mean (SD)**	**Acupressure (n=50)** **Mean (SD)**	**Control (n=50)** **Mean (SD)**	**Total (n=150)** **Mean (SD)**
**Unobserved**	127.0 (52.0)	212.2 (49.1)	337.8 (68.9)	224.4 (61.5)
**Observed**	227.7 (33.7)	242.5 (56.9)	417.7 (107.1)	361.6 (125.3)

*P. *value<0.01; According to logistic regression analysis and eliminating the effect of group [OR=1.009; 95% CI (1.002-1.017)]

NRBC: Nucleated red blood cells; SD: Standard deviation

After the intervention, the anxiety score decreased by 20.5 points in the acupressure group and by 20 points in the supportive care group, but increased by 11.1 points in the control group. Chao et al (2007) also performed acupressure at various points and revealed this non-pharmacological method to be effective in reduction of women's pain and anxiety during labor^[^^21^^]^. In the same line, the results of the study by Fassoulaki et al (2003) showed the effectiveness of acupressure in reduction of pain and stress^[^^22^^]^. These results were in line with those of the present study in which, the anxiety score decreased by 34.8% in the acupressure group after the intervention. Considering the supportive care group, the present study results were in agreement with those of the study by Hofmeyr et al (1991) reporting the anxiety mean score as 28.2 in the supported group and 37.8 in the routine care group^[^^23^^]^. In the current study also, the mean score of anxiety reduced by 37.5% in the supportive care group after the intervention which might be due to accompanying and supporting the mother leading to reduction of her pain and anxiety. Pilkington et al (2007) also expressed the effect of acupressure on the patients’ anxiety level based on Spielberger’s scale^[^^24^^]^. Various studies have shown that acupressure, without any complications, controls and reduces anxiety by stimulating brain responses and hormonal activities through increasing the blood flow and mediating the metabolism^[^^25^^]^. Therefore, it can be concluded that mother’s anxiety which is accompanied by increased uterine vessels' resistance and decreased oxygenation plays a major role in fetal as well as maternal outcomes. This emphasizes the necessity for performing interventions through labor in order to reduce the mothers’ anxiety. On the contrary, Langer et al (1998) showed that supporting the women through labor was not effective in the need for medical interventions and mothers' anxiety, pain, self-confidence, and satisfaction. In that study, the mean score of anxiety was 49.1 in the supported group and 49.2 in the control group and the difference was not statistically significant^[^^26^^]^. The difference between that study and the present one might be due to intervention methods, hospital policies, women's cultural background, short period of support, and types of doulas' activities. Yet the support provided by the nurses and the staff might have also been effective in reducing the difference between the two groups.

 In this study, distribution of the NRBCs in the peripheral blood smears was 18%, 20%, and 86% in the supportive care, acupressure, and control groups, respectively. Stress and anxiety lead to secretion of epinephrine. 

**Table 4:** The relationship between the frequency of NRBC in the cord blood and delivery mode in the intervention (supportive care and acupressure) and control groups

**Delivery mode**	**NRBC in cord blood count, ** **peripheral smear**	**Intervention (n=100)** **n (%)**	**Control (n=50)** **n (%)**
**Vaginal delivery**	**Unobserved**	80 (86.0)	7 (23.3)
	**Observed**	13 (14.0)	23 (76.7)
**Caesarean section**	**Unobserved**	1 (14.3)	0
	**Observed**	6 (85.7)	20 (100)

*P.* value<0.001 in intervention group and 0.3 in control group; NRBC: Nucleated red blood cells

Epinephrine through beta-adrenergic receptors in the uterus leads to uterine muscle hypoxia, disruption in uteroplacental blood perfusion, and fetal hypoxia^[^^27^^]^. Basically, intrauterine hypoxia is one of the main factors in increasing erythropoietin which stimulates the fetal hematopoietic system and increases the production of NRBCs. Since NRBCs can change their shape, size, and expansibility, they are released from the bone marrow into the peripheral blood. In the current study, the number of the cases with NRBCs detected in the peripheral blood smears was higher in the control group compared to the two intervention groups. Thus, fetal hypoxia was probably higher in the control group which received no interventions for reduction of pain and anxiety during labor. Several studies have indicated a significant relationship between the number of NRBCs and fetal hypoxia and have considered the number of NRBCs as an important index for identifying hypoxia^[^^28^^-^^30^^]^. Predicting the importance of fetal hypoxia needs awareness of its duration and grade^[^^31^^]^. NRBC count increases in acute and chronic hypoxia but the more the fetal hypoxia, the more increases NRBC count^[^^29^^]^. The average rate of nucleated red blood cells is 500 in mm^3^ in the first hours of healthy term infant’s life (more than 1000 is abnormal)^[^^32^^]^. 1 to 2 percent of healthy infants have increased nucleated red blood cells without any reason^[^^33^^]^. For instance, in the study by Tomar et al (2011), the mean number of NRBCs was 10.34+3.87 in the fetal distress group and 5.7+2.33 in the control group^[^^34^^]^. In addition, Hanion-Lundberg et al (1999) measured the mean number of NRBCs as 9.2+18.1 in 1561 hypoxic infants^[^^35^^]^. In the study by Kovalak et al (2011) the mean number of NRBCs was 13 (range: 0-37) in the case group and 8 (range: 0-21) in the control group^[^^36^^]^. In the present study, providing the mothers with doula supportive care and applying pressure at BL32 acupoint were performed to decrease the effective factors in fetal hypoxia including anxiety, and prevent the undesirable fetal and maternal outcomes. However, no such interventions were carried out in the studies by Hanion-Lundberg and Kovalak. 

 In Hanion-Lundberg’s study, NRBC count in infants with first minute’s Apgar 0-3 was significantly and statistically higher than that in infants with Apgar >7; this study included also infants with diabetic mothers and meconium stained cases. In our study, these cases and also infants with basis problem were excluded and pregnancy was terminated immediately after detection of abnormal heart models. Also, ill neonates with low Apgar that needed NICU were not in our study. Our neonates despite the abnormal heart rate model in control group had Apgar scores higher than 7 and did not need resuscitative measures and NICU care, and distress and intrauterine hypoxia intensity and duration were not high enough to cause over increase in NRBC count.

 In this study, the labor was longer in the cases with fetal NRBCs detected in the peripheral blood smears. Besides, the highest and lowest labor length was related to the control (417.7+107.1) and supportive care group (227.7+33.7), respectively. As the labor length increases, mother's anxiety increases, as well. Thus, due to secretion of stress hormones, including cortisol, epinephrine, and norepinephrine, the vessels are contracted and less oxygen is delivered to the fetus. Then, the production of erythropoietin is increased in response to hypoxia and NRBCs enter the peripheral blood. A large number of studies have also shown that the infants exposed to intrauterine hypoxia have more NRBCs in their cord blood ^[^^28^^-^^30^^,^^37^^,^^38^^]^. In the studies by Ferns^[^^39^^]^ and Lim^[^^40^^]^ also, a significant correlation was found between the length of labor and the number of NRBCs, which is consistent with the findings of the present study. However, Kovalak^[^^36^^]^ reported no significant relationship between the number of NRBCs and the length of labor. This difference might result from the fact that most of the control group women in Kovalak's study underwent cesarean section and, consequently, the length of labor was shorter in the control group compared to the case group (distressed infants). In contrast to the current study, Ghosh^[^^8^^]^ did not consider hypertension, preeclampsia, tobacco use, meconium, growth restriction, and chorioamnionitis which can increase the number of NRBCs as the exclusion criteria.

 In this study, a larger number of NRBCs were detected in peripheral blood smears in cesarean delivery in all the three groups. This implies a significant relationship between the type of delivery and the number of NRBCs in the blood cord in both the intervention groups and the control group. In line with these results, Ferns^[^^39^^]^ also believed that the number of NRBCs was affected by the delivery mode. On the contrary, in the study conducted by Saracoglu et al (2000), the mean number of NRBCs was 8.25+3.39 and 7.58+4.04 in the cesarean and natural delivery, respectively and the difference was not statistically significant^[^^41^^]^. Kovalak^[^^36^^]^, Axt^[^^33^^]^, Ghosh^[^^8^^]^, Hanion-Lundberg^[^^35^^]^, and Korst^[^^28^^]^ also found no significant relationship between the number of NRBCs and type of delivery. This might be due to the fact that the above-mentioned studies had not exclude hypertension, preeclampsia, tobacco use, meconium, growth restriction, and chorioamnionitis. These factors increase the number of NRBCs regardless of the delivery mode and labor length because by uterine vessels contraction in these patients, perfusion and oxygenation to the fetus is decreased and more NRBCs are produced and entered into the peripheral blood in response to hypoxia.

## Conclusion

The findings of the present study showed that supportive care and acupressure were effective, safe, simple, and inexpensive techniques which could be used in order to reduce the delivery pain, mother’s anxiety, and length of labor, resulting in improvement of oxygenation to the fetus and prevention of asphyxia. Asphyxia, regardless of duration, does not always increase the NRBC count and NRBC count is far more increased in some cases without asphyxia. Therefore, NRBC count is not the only decisive factor of intrauterine asphyxia intensity and duration. In case a large number of NRBCs are detected in infants, they should be provided with more care in order to prevent them from probable nervous damages. Although the number of NRBCs alone cannot be a prognostic factor, further studies on this issue can confirm or reject the results obtained in the current study. Overall, considering the national and international approach toward physiological delivery, this non-pharmacological method is recommended to be used to reduce the mothers' anxiety during labor and improve the maternal and fetal outcomes.

 One of the limitations of this study was interference of gynecologists and midwives in the normal process of labor. In case their interventions affected the results, the patient was excluded from the study. Moreover, the participants’ mental conditions while completing the questionnaire, family problems, and undiagnosed diseases could affect the study results, which was out of the researcher’s control.

## Authors’ Contribution

Z. Masoudi, M Akbarzadeh, F. Vaziri and M. Ramzi conceived of the study and participated in its design and coordination and helped to draft the manuscript. Z. Masoudi collected the data. Z. Masoudi and N. Zare performed the statistical analysis. Z. Masoudi and M. Akbarzadeh made critical revisions to the paper and translated it into English language. All authors read and approved the final version of the paper.

## References

[1] Simkin PP, O'Hara M (2002). Nonpharmacologic relief of pain during labor: systematic reviews of five methods. Am J Obstet Gynecol.

[2] Hall WA, Hauck YL, Carty EM (2009). Childbirth fear, anxiety, fatigue, and sleep deprivation in pregnant women. J Obstet Gynecol Neonatal Nurs.

[3] Arai YC, Ueda W, Ushida T (2009). Increased heart rate variability correlation between mother and child immediately pre-operation. Acta Anaesthesiol Scand.

[4] Arai YC, Ueda W, Ito H (2008). Maternal heart rate variability just before surgery significantly correlated with emergence behavior of children undergoing general anesthesia. Paediatr Anaesth.

[5] Maimburg RD, Vaeth M, Durr J (2010). Randomised trial of structured antenatal training sessions to improve the birth process. BJOG.

[6] Lawn JE, Cousens S, Zupan J (2005). 4 million neonatal deaths: when? Where? Why?. Lancet.

[7] Martin RJ, Fanaroff AA, Walsh MC ( 2006). Neonatal Perinatal Medicine.

[8] Ghosh B, Mittal S, Kumar S (2003). Prediction of perinatal asphyxia with nucleated red blood cells in cord blood of newborns. Int J Gynecol Obst.

[9] Fink NS, Urech C, Isabel F (2011). Fetal response to abbreviated relaxation techniques. A randomized controlled study. Early Hum Dev.

[10] Kimber L, McNabb M, Mc Court C (2008). Massage or music for pain relief in labour: A pilot randomised placebo controlled trial. Eur J Pain.

[11] Zhang J, Bernasko JW, Leybovich E (1996). Continuous labor support from labor attendant for primiparous women: a meta-analysis. Obstet Gynecol.

[12] Teixeira JM, Fisk NM, Glover V (1999). Association between maternal anxiety in pregnancy and increased uterine artery resistance index: cohort based study. BMJ.

[13] Reynolds F (2010). The effects of maternal labour analgesia on the fetus. Best Pract Res Clin Obstet Gynaecol.

[14] Lee MK, Chang SB, Kang DH (2004). Effects of SP6 acupressure on labor pain and length of delivery time in women during labor. J Altern Complement Med.

[15] Caton D, Corry MP, Frigoletto FD (2002). The nature and management of labor pain: executive summary. Am J Obstet Gynecol.

[16] May AE, Elton CD (1998). The effects of pain and its management on mother and fetus. Baillière's Clin Obst Gynaecol.

[17] Cook A, Wilcox G (1997). Pressuring pain. Alternative therapies for labor pain management. AWHONN Lifelines.

[18] Spielberger CD, Gorsuch RL, Lushene R (1983). Manual for the State-Trait Anxiety Inventory.

[19] Aghamohammadi M (2008). Religious preoperative anxiety. Ann Gen Psychiat.

[20] Hadianfard M, Shokrpour N (2011). Acupoints In illustrated tables.

[21] Chao A-S, Chao A, Wang T-H (2007). Pain relief by applying transcutaneous electrical nerve stimulation (TENS) on acupuncture points during the first stage of labor: A randomized double-blind placebo-controlled trial. Pain.

[22] Fassoulaki A, Paraskeva A, Patris K (2003). Pressure applied on the extra 1 acupuncture point reduces bispectral index values and stress in volunteers. Anesth Analg.

[23] Hofmeyr GJ, Nikodem VC, Wolman W (1991). Companionship to Modify the Clinical Birth Enviorment: Effects on progress and perceptions of labour and breast feeding. Br J Obstet Gynecol.

[24] Pilkington K, Kirkwood G, Rampes H (2007). Acupuncture for anxiety and anxiety disorders - a systematic literature review. Acupunct Med.

[25] Edelman M, Ficorelli C (2005). A measure of success: nursing students and test anxiety. J Nurses Staff Dev.

[26] Langer A, Campero L, Garcia C (1998). Effects of psychosocial support during labour and childbirth on breastfeeding, medical interventions, and mothers' wellbeing in a Mexican public hospital: a randomised clinical trial. Br J Obstet Gynaecol.

[27] Campell DA, Lake MF, Falk M (2006). A randomized control trial of continuous support in labour by a lay doula. J Obstet Gynecol Neonatal Nurs.

[28] Korst LM, Phelan JP, Ahn MO (1996). Nucleated red blood cells: an update on the marker for fetal asphyxia. Am J Obstet Gynecol.

[29] Phelan JP, Ahn MY, Korst LM (1995). Nucleated red blood cells: a marker for fetal asphyxia?. Am J Obstet Gynecol.

[30] Thilaganathan B, Athanasiou S, Ozmen S (1994). Umbilical cord blood erythroblast count as an index of intrauterine hypoxia. Arch Dis Child Fetal Neonatal Ed.

[31] Low JA (1988). The role of blood gas and acid-base assessment in the diagnosis of intrapartum fetal asphyxia. Am J Obstet Gynecol.

[32] Hanion-Lundberg KM, Kirby RS, Gandhi S (1997). Nucleated red blood cells in cord blood of singleton term neonates. Am J Obstet Gynecol.

[33] Axt R, Ertan K, Hendrik J (1999). Nucleated red blood cells in cord blood of singleton term and post-term neonates. J Perinat Med.

[34] Tomar G, Sikarwar S, Gupta S (2011). The correlation of clinical perinatal asphyxia with counts of Nrbc/100 WBC in cord blood. Webmed Central Obs Gyn.

[35] Hanion-Lundberg KM, Kirby RS (1999). Nucleated red blood cells as a marker of acidemia in term neonates. Am JObstet Gynecol.

[36] Kovalak EE, Dede FS, Gelisen O (2011). Nonreassuring fetal heart rate patterns and nucleated red blood cells in term neonates. Arch Gynecol Obstet.

[37] Philip AG, Tito AM (1989). Increased nucleated red blood cell counts in small for gestational age infants with very low birth weight. Am J Dis Child.

[38] Soothill PW, Nicolaides KH, Campbell S (1987). Prenatal asphyxia, hyperlacticaemia, hypoglycaemia, and erythroblastosis in growth retarded fetuses. Br Med J (Clin Res Ed).

[39] Ferns SJ, Bhat BV, Basu D (2004). Value of nucleated red blood cells in predicting severity and outcome of perinatal asphyxia. Indian J Pathol Microbiol.

[40] Lim FT, van Winsen L, Willemze R (1994). Influence of delivery on numbers of leukocytes, leukocyte subpopulations, and hematopoietic progenitor cells in human umbilical cord blood. Blood Cells.

[41] Saraçoglu F, Sahin I, Eser E (2000). Nucleated red blood cells as a marker in acute and chronic fetal asphyxia. Int J Gynecol Obstet.

